# Ontology-driven integrative analysis of omics data through Onassis

**DOI:** 10.1038/s41598-020-57716-1

**Published:** 2020-01-20

**Authors:** Eugenia Galeota, Kamal Kishore, Mattia Pelizzola

**Affiliations:** Center for Genomic Science of IIT@SEMM, Fondazione Istituto Italiano di Tecnologia, Milano, Italy

**Keywords:** Data integration, Software

## Abstract

Public repositories of large-scale omics datasets represent a valuable resource for researchers. In fact, data re-analysis can either answer novel questions or provide critical data able to complement in-house experiments. However, despite the development of standards for the compilation of metadata, the identification and organization of samples still constitutes a major bottleneck hampering data reuse. We introduce Onassis, an R package within the Bioconductor environment providing key functionalities of Natural Language Processing (NLP) tools. Leveraging biomedical ontologies, Onassis greatly simplifies the association of samples from large-scale repositories to their representation in terms of ontology-based annotations. Moreover, through the use of semantic similarity measures, Onassis hierarchically organizes the datasets of interest, thus supporting the semantically aware analysis of the corresponding omics data. In conclusion, Onassis leverages NLP techniques, biomedical ontologies, and the R statistical framework, to identify, relate, and analyze datasets from public repositories. The tool was tested on various large-scale datasets, including compendia of gene expression, histone marks, and DNA methylation, illustrating how it can facilitate the integrative analysis of various omics data.

## Introduction

The plummeting cost of high-throughput sequencing experiments has led to a rapid accumulation of omics datasets in public repositories. For example, more than 600 K RNA-seq and 80 K ChIP-seq samples are currently available in Gene Expression omnibus (GEO^1^) and Sequence Read Archive (SRA^2^). However, most published studies covering few regulatory factors and/or epigenetic marks profiled over a limited number of biological conditions rely on newly generated omics data, thus ignoring the fact that massive collections of publicly available data could be exploited to study those very same factors and their interactions in identical or very similar conditions. Data re-use is mainly hindered by technical challenges in data retrieval, analysis and integration. In particular, biologists have little familiarity with the necessary tools^3^, which are seldom user friendly. Moreover, despite the introduction of standards such as MINSEQE and MIAME^4^, sample metadata are typically highly heterogeneous thus making samples retrieval complicated. Available integrative approaches are oriented to the development of public accessible interfaces^5^ where users can query databases for their data of interest or download pre-compiled datasets. However, these are typically limited in terms of the available datasets, strongly depend on the willingness and ability of the developers to maintain and update the system and the available data. Ultimately, these solutions offer a fixed, pre-defined set of functionalities, and as a result, they are suboptimal in terms of flexibility. Instead, federated databases use semantic web technologies^5^ to represent their data in RDF (Resource Description Format) to be simultaneously queried using the language SPARQL. In this case the drawback is that knowledge of a very specific query language is needed to make proper queries. More advanced methods, like heterogeneous information networks^6^, provide data structures and analytical methods to handle data heterogeneity in integrative analyses. However, these methods require solid computational skills to correctly develop and mine such networks. Finally, key repositories, like GEO, are not represented in RDF format and exploratory queries can be tricky.

Ontologies play a key role in facilitating the exploitation of publicly available datasets. They are widely applied to support semantic- and content-based information extraction in domains of interest^7–9^ and the concepts they contain can be used as dictionaries to annotate datasets metadata using standardized identifiers^10–12^. Moreover, relationships between ontology concepts can be used to structure a dataset according to how its samples’ metadata semantically relate to each other^12,13^. However, the use of biomedical ontologies is typically restricted to the computer science domain, and with the exclusion of the popular Gene Ontology, they rarely reach the community of biologists, while this would greatly benefit from their support.

With the aim of lowering the barrier to data reuse, we developed Onassis (Ontology Annotations and Semantic Similarity Software), a tool that leverages NLP techniques, biomedical ontologies, and the R statistical framework, to identify, relate, and analyze datasets from public repositories. First, with a process known as named entity recognition, Onassis associates free textual descriptions of publicly available samples to the concepts belonging to ontologies where entities of a given domain of interest are associated to a standard representation. Second, semantic similarity measures, quantifying the similarity of ontology concepts based on the underlying structure of ontological relationships, can be used by Onassis to determine the closeness of samples based on their ontological annotations. Finally, statistical tests with any custom function can be effortlessly applied within Onassis to analyze the corresponding omics data, driven by the semantic aware samples organization.

## Onassis Description

**Figure 1:**
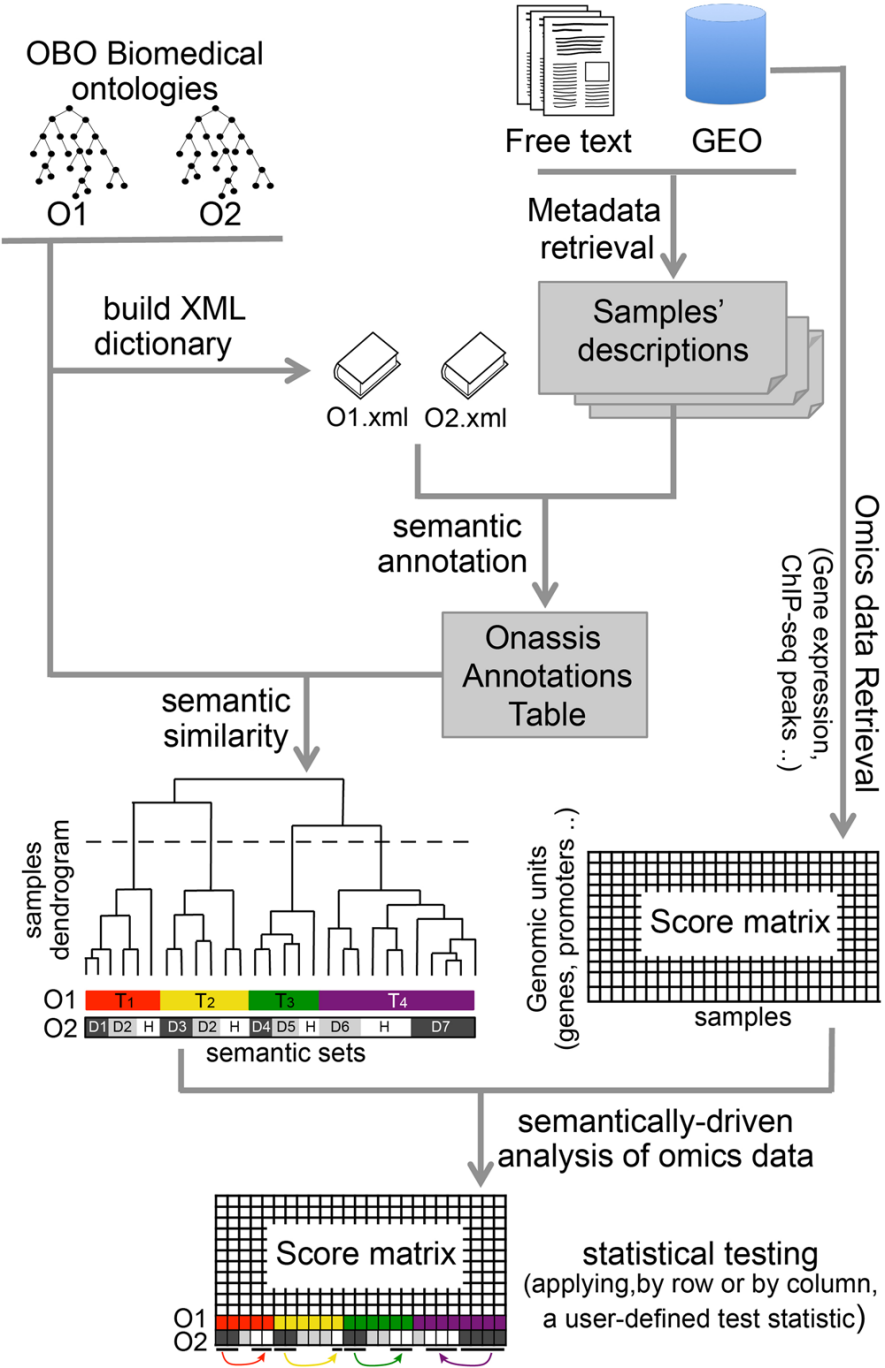
Onassis workflow. Samples metadata are retrieved from various data sources such as GEO, journals’ abstracts or any free text. OBO ontologies are adopted to create a dictionary for a given domain of interest (for example tissues and/or diseases). Samples descriptions are associated with ontology concepts through NLP tools, and semantic similarities are quantified, providing a hierarchy of samples based on their similarity; T_1–4_ and D_1–7_ denote four different tissue conditions and seven different disease conditions, respectively, while H indicates healthy samples for each tissue. The organization of samples by semantic annotations and relationships are used to drive the analysis of the actual omics data, thus testing for differences between the scores in different semantic sets. For example, the arrows indicate all pairwise comparisons of a disease with the healthy counterpart. This would return, “by row”, the set of genomic units differential in the score; instead, “by column”, this would return the global difference between the samples associated with the compared conditions.

Onassis is available as a package within the R/Bioconductor project^14^, a very popular software repository for the analysis of genomic data, used by both bioinformaticians and biologists. The package functionalities assist users in performing the following tasks (Fig. 1):

- building a dictionary with concepts from Open Biomedical Ontologies (OBO)^15^
- retrieving metadata associated with collections of GEO samples (dataset)
- configuring and running a named entity recognition pipeline to annotate retrieved metadata (or any collections of textual documents) with the previously built dictionary, therefore associating them with ontology concepts
- structuring the dataset of interest by hierarchically organizing its samples according to the semantic similarity between their associated ontology terms
- performing a semantically-driven statistical analysis of the actual omics data in the dataset.

### Input metadata

Metadata can be retrieved from large-scale repositories of omics data, such as GEO and SRA. In particular, the metadata of GEO samples, a reference repository in the field, can be conveniently accessed in Onassis via GEOmetadb^16^. This package stores the metadata of GEO samples and datasets in an SQLite database, with tables containing information such as the title of the experiment, the list of samples, their descriptions, details about the organism, sequencing libraries, description of treatments, and other attributes. While queries through SQLite statements are permitted, as originally intended in GEOmetadb, Onassis implements simplified functions to easily query and subset the metadata tables avoiding SQLite statements. For a proper annotation, particularly important is the choice of the fields to be passed to the annotator^12^. A visual inspection of GEOmetadb tables revealed that the most informative fields for the description of experiments are title and summary, while for samples source_name_ch1 for sample source, organism_ch1, characteristics_ch1 and description were selected. Finally, filters are provided for the type of platform (e.g. Illumina HiSeq 2000), experiment (e.g. ChIP-seq, RNA-seq), and organism. Ensuring the full flexibility, metadata from any other resource can be provided to Onassis in textual files.

### Named entity recognition

The process of text annotation using controlled vocabularies relies on the ccp-nlp implementation of Conceptmapper^17^, which we and others previously showed that outperforms other available tools^12,18^. XML dictionaries, required by Conceptmapper, can be automatically generated by Onassis starting from OBO ontologies (in both OBO v1.2 and OWL formats). Entries of the dictionary are specified by a canonical name and one or more variants (synonyms).

The annotation pipeline consists of a sequence of steps: detection of sentences in the metadata, tokenization of both metadata and individual dictionary entries which can be composed of multiple words (tokens), optional stemming of the tokens to reduce word inflections and lookup of the dictionary tokens within sentences. When it comes to building dictionaries and named entity recognition pipelines, various configuration parameters can be set, including: the type of synonyms to include in the dictionary (broad, narrow, related or exact as defined in the OBO rules), case sensitivity, stop words removal to ignore uninformative tokens, stemmers, different lookup strategies to consider ordered tokens or their permutations, finding all possible matches or only the longest, allowances for overlaps between subsequent matches^19^. Eventually, duplicated annotations are removed and a table associating each sample with a list of unique ontology concepts (that we define as semantic set) is returned.

Importantly, configuration parameters were set to default values based on recent benchmarking studies^12,18^. In particular, in the former, we evaluated the performances of applying Conceptmapper to GEO metadata based on the Brenda^20^ and Disease Ontology^21^, to associate GEO samples to specific tissues and disease conditions. The accuracy of Conceptmapper was also compared to the performance of Metamap^22^, another popular tool in the field. As a result, Conceptmapper reached an accuracy of 0.8 and 0.9, for the disease and tissue ontologies, respectively, while Metamap was in the 0.56–0.65 range^12^.

Finally, in order to identify in the metadata terms such as gene/protein names and epigenetic modifications, Onassis can automatically build dictionaries for those terms, using Bioconductor libraries available for multiple species. This is particularly useful for example to associate the metadata of ChIP-chip or ChIP-seq experiments to the specific targeted factors.

### Semantic similarity

Once samples of large dataset are associated to concepts of a biomedical ontology, it could become evident that the various experiments are not independent. Indeed, the associated concepts are likely to reveal samples that can be considered equivalent or very similar, and others that can be considered far from each other. In other words, relations between samples emerge, which can give a structure to the dataset and can be quantified. Indeed, the relationships encoded in an ontology structure can be used to quantify the semantic similarity between its concepts^13^. Several tools for the computation of semantic similarities have been proposed, including GOSemSim^23^ and DOSE^24^, which are tailored on Gene Ontology and Human Disease Ontology, respectively. Their functions allow for the calculation of functional similarity between genes and gene groups, but are limited on a single ontology, implement only a limited number of similarity measures and rely on Bioconductor annotation databases, which are not available for every OBO ontology. Another package, MeSHSsim^25^ allows the computation of semantic similarities between MeSH headings, but this is tailored for MEDLINE documents.

In Onassis, samples annotated with one or more ontologies can be related to each other based on the semantic similarity between their associated semantic sets (i.e. sets of associated concepts). Pairwise samples similarities can be used for their hierarchical clustering, and samples associated with similar semantic sets (based on a user-defined cutoff of similarity) can be grouped (collapsed) into semantically homogenous clusters. For large datasets this can be important to reduce the complexity of resulting dendrogram, and to increase the number of observations (samples) which can be brought back to a common semantic set.

Various similarity measures are available within Onassis by wrapping functions from the Semantic measures library and toolkit^26^. Specifically, the ontology, represented as a graph, can be traversed to calculate the semantic similarity between pairs (pairwise similarity) or groups (group-wise similarity) of concepts. This is important for determining the similarity between samples that can be easily associated to multiple concepts. Noteworthy, since most of the semantic similarity measures consider only one type of edge and given that “part-of” relationships are widely used in current ontologies, we converted them into the most common “is-a” type. Finally, Onassis allows users to combine similarities from multiple ontologies by using any aggregating function of choice (such as mean, median or more complex functions). The choice of the semantic similarity measure can influence the way in which samples are combined. However, difficulties in assessing the performances of semantic similarities arise due to the lack of a gold standard. In fact, the performance of these measures is expected to be highly dependent on the specific task and on the features of the considered corpus, such as annotation size and depth or specificity of annotation classes^27,28^.

### Semantically-driven statistical analysis of samples omics data

Once the semantic information is associated to the samples (based, for example, on the annotation of samples metadata with cell lines and disease conditions), Onassis uses it within the *compare* function, in order to direct the analysis of the actual omics data (Fig. 1).

This requires that the omics data are stored within a score matrix, whose rows represent genomic units and whose columns represent samples. We reasoned that the two most likely genomics unit would be either genes or genomic regions. In the former case, the score may be any omics data that can be associated with individual genes, such as gene-level RNA-seq expression data. In the latter case, the score may be any omics data that can be associated with individual genomic regions, such as the enrichment of ChIP-seq for a given factor or mark within each promoter.

Onassis takes then advantage of the semantic information to compare these scores among samples. Specifically, the semantic similarity determines the grouping of the semantic sets, and the scores will be compared between those sets. The function used for comparing the scores could be any of the R-statistical tests, typically returning a test statistic and a multiple-testing corrected p-value. Importantly, users are allowed to define and use their own personalized test function. Tests can be applied by columns, for a pair-wise comparison of semantic sets based on global scores distributions. Instead, the comparison by rows perform the analysis at the level of each genomic unit, leading to the identification of units that are differential among the semantic sets. Finally, in case samples were annotated using two different ontologies, such as cell types and disease ontologies, the corresponding semantic sets can be passed on to Onassis simultaneously. This enables Onassis to perform the above-mentioned analyses by automatically iterating over the semantic sets defined on the primary ontology (e.g. tissue types), while comparing, by row or column, the scores among the states defined on the secondary ontology (e.g. disease conditions identified for each tissue condition). The following use cases will illustrate these analyses in detail.

## Use Cases

We adopted Onassis for the integrative analysis of large-scale omics datasets in three different contexts: (i) a large dataset of H3K27ac ChIP-seq samples; (ii) a compendia of 620 RNA-seq samples from GEO; (iii) a large-compendia of ~15 K DNA-methylation samples. These analyses, which are presented below, illustrate how Onassis functionalities can be used for a semantically-aware analysis of omics data.

### H3K27ac marks across tissues and diseases

**Figure 2:**
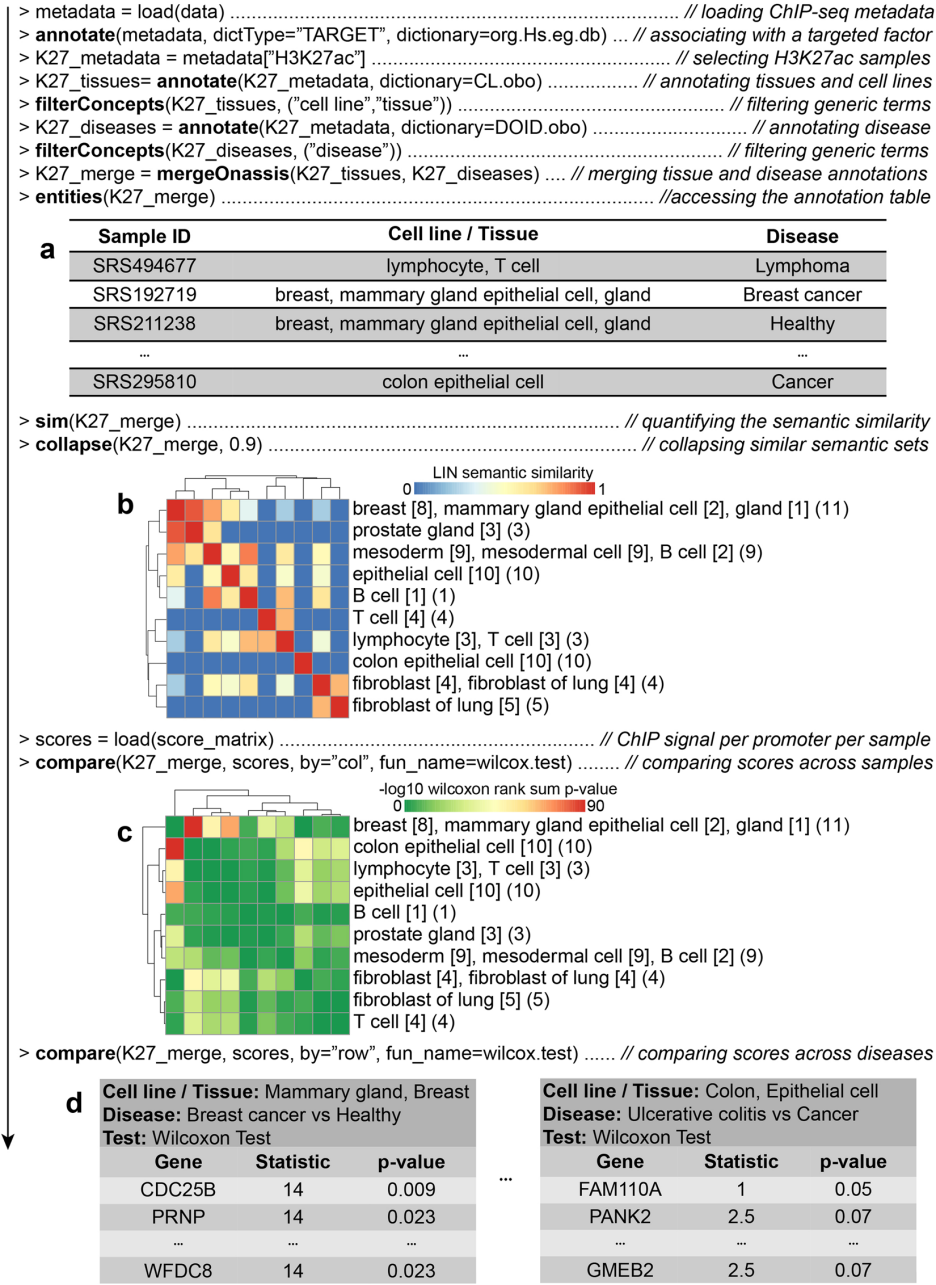
Integrative analysis of H3K27ac ChIP-seq samples through Onassis. The key steps of an analysis with Onassis are shown as pseudocode, each line commented on its right-end. Package functions are indicated in bold. (**a**) An extract of the table returned by the *entities* function, which contain for each sample the associated concepts. (**b**) Heatmap of the semantic sets organized according to their semantic similarity (1 being the maximum similarity). Each semantic set combines one or more samples, the number is indicated in brackets at the end. For example, the first semantic set combines 11 samples. Instead, the number of samples associated with each concept is reported in squared brackets beside it. For example, 8 out of 11 samples in the first semantic set were associated with the concept “breast”. (**c**) Heatmap displaying the global difference in H3K27ac between pairs of semantic sets. (**d**) Heatmap displaying, for each tissue type, the set of genes whose promoter are differential in H3K27ac. Tests were conducted comparing each pair of disease semantic sets associated with the given tissue. For example, CDC25B was found comparing Breast cancer vs. the healthy counterpart in the “Mammary gland, Breast” tissue semantic type. Test statistics and the corresponding p-values are reported. The full code for this analysis is available within the Onassis Bioconductor vignette, in the section documenting the Onassis class.

H3K27ac epigenetic modifications are commonly found in proximity of active promoters, and co-localize with active tissue-specific enhancers^29^. As illustrated by Fig. 2, we used Onassis to explore the distribution of H3K27ac marks in the context of various cell lines/tissues and disease conditions. Metadata related to ChIP-seq samples were retrieved from SRAdb^30^, a database describing experiments and samples in SRA, the main public repository for high throughput sequencing data. Similarly to GEOmetadb, SRAdb can be explored through SQLite queries. Metadata of human ChIP-seq samples were obtained by querying the database according to library strategy, library source and organism attributes of experiments. The text from informative fields, which included information about the target of the ChIP-seq experiment, cell type or tissue and disease conditions (if any), was passed as input to Onassis. H3K27ac samples metadata were thus annotated with Cell Ontology^31^ (CL) and Human Disease Ontology^21^ (DOID) concepts. The default parameter configuration was adopted for the annotation pipeline, since it is the best performing according to previous analyses^12,18^. Generic or uninformative terms such as ‘cell line’ or ‘disease’ were filtered out, and cell line/tissue and disease semantic sets were defined for each sample (Fig. 2a). Finally, samples belonging to very similar semantic sets, with LIN similarity (the best performing similarity measure according to previous studies^12^) values higher than 0.9 were associated to a unique tissue semantic set (Fig. 2b).

The ChIP-seq peaks corresponding to the annotated samples were retrieved from Cistrome^32^, a project aimed at facilitating the integrative analyses of omics data by providing uniformly re-analyzed genomics data for human and mouse. H3K27ac peaks were summarized into a score matrix, having as rows promoter regions and as columns samples identifiers. Entries of the matrix in which a given promoter (row) was bound by the mark in a specific sample (column) were filled with the corresponding peak intensities. The resulting score matrix and the annotations were fed into Onassis *compare* function, to automatically apply two-tailed Wilcoxon rank sum tests, thus revealing global differences in H3K27ac within promoters between pairs of diseases for each tissue semantic set (Fig. 2c). The pseudocode reported in Fig. 2 illustrates that these analyses can be performed with a simple set of instructions, requiring only basic knowledge of R.

We then focused on the identification of individual promoters, differential in H3K27ac among the diseases within each tissue type. The Fig. 2d reports for example the genes downstream the promoters identified when breast cancer samples were compared to the healthy counterpart (Fig. 2d). Noteworthy, this analysis returned the CDC25B gene, known to be involved in breast cancer and being a potential therapeutic target^33^.

**Figure 3:**
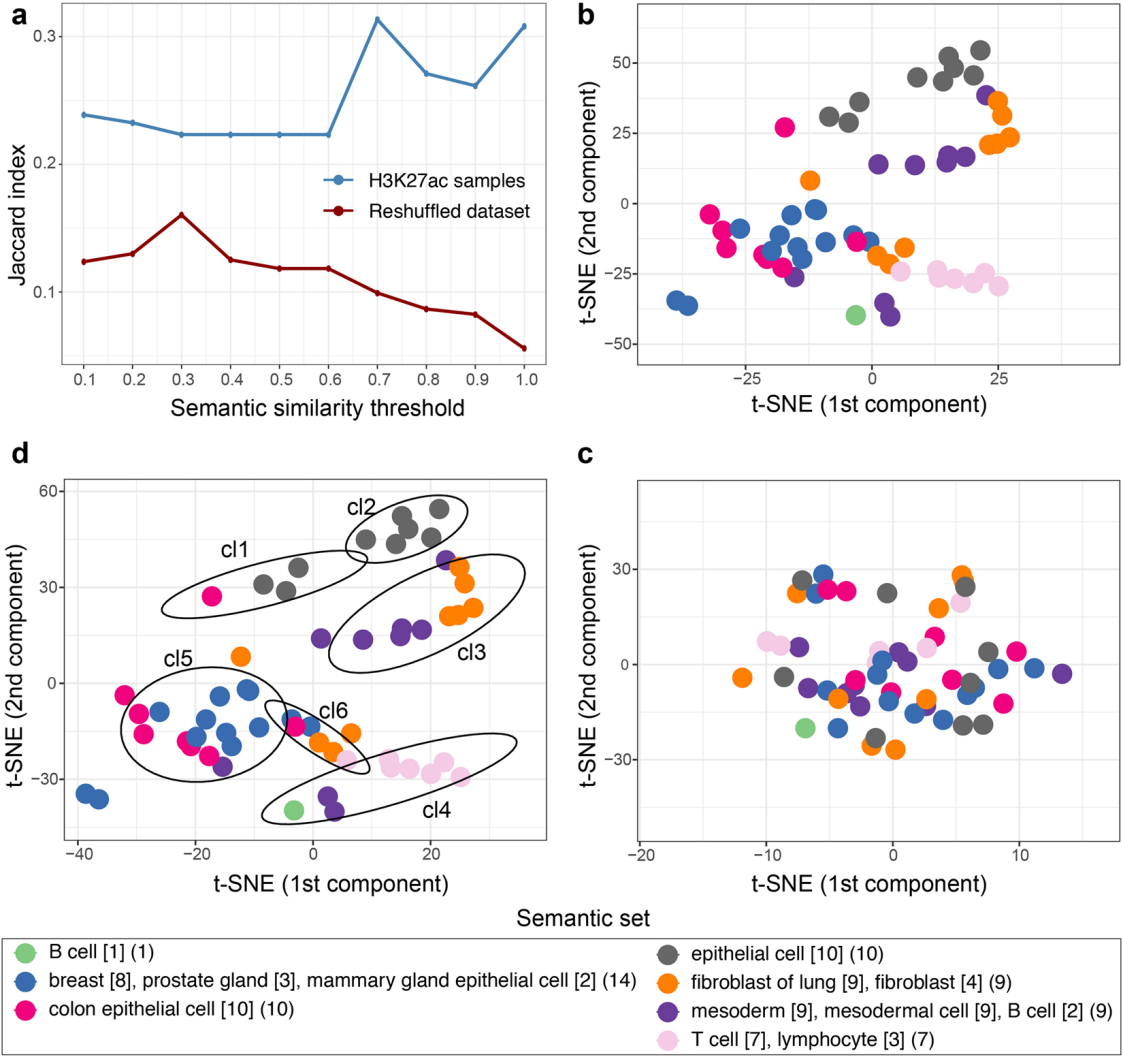
Correspondence between semantically-driven and data-driven analyses. (**a**) The Jaccard index is used to quantify the overlap between semantically-driven (determined through Onassis) and data-driven (determined through the t-SNE dimensionality reduction method) H3K27ac samples groups. Semantic groups were identified at a given semantic similarity threshold (x-axis), and compared to an equal number of data-driven groups. The overlap analysis was repeated following the reshuffling of the genomics data. (**b**) t-SNE plot of genome-wide H3K27ac densities at promoters. Each sample was color-coded based on the semantic grouping obtained at similarity threshold of 0.7. Semantic sets are described in the legend at the bottom of the figure, using the notation described in Fig. 2b. (**c**) As in (**b**) following the reshuffling of the genomics data. (**d**) as in (b) with the addition of the data-driven clusters (cl), see discussion in the text.

We reasoned that the semantic similarity among the H3K27ac samples should reflect, at least in part, the similarity of the actual ChIP-seq data. To this end, we first identified semantic groups at different cutoff values of semantic similarity. For each combination of semantic groups, we then used the t-SNE dimensionality reduction method to identify an equal number of data-driven groups, based on promoter H3K27ac densities. The overlap between semantic and data-driven groups was quantified with the Jaccard index. The overlap analysis was then repeated using a randomly reshuffled score matrix. As expected, this disrupted any association between samples metadata and epigenomics data, leading to lower Jaccard indexes (Fig. 3a). For one of these combinations (semantic similarity cutoff at 0.7), the color-coded semantic grouping was combined with the data-driven positioning of samples in the t-SNE plot (Fig. 3b). The proximity of samples associated with the same semantic group indicates that they have similar patterns of H3K27ac at promoters. In addition, this association is lost following the reshuffling of the score matrix, since the color-coded semantic grouping is markedly mixed (Fig. 3c). Finally, data-driven and semantic groups were overlaid (Fig. 3d). While not all data-driven clusters appear homogenous from the semantic point of view, the associated tissue types are closely related. Indeed, clusters 1 and 2 (cl1 and cl2) include mostly epithelial cells not assigned to specific tissues or organs. Clusters 3 and 4 include mostly cell types of mesodermal origin (lung fibroblasts in cl3, and blood cells in cl4). Cluster 5 includes mostly breast and colon epithelial cells. Altogether, there is a good correspondence between the semantic and data-driven analyses, while, as expected, specific groups can be identified that are prevalently driven by similarity in terms of genomics data or tissue type.

The integration of Onassis within the Bioconductor project greatly facilitates downstream analyses such as those presented here, due to the broad offer of packages devoted to the analysis of omics data^34^. Importantly, this also justifies the acquisition of the required basic R skills.

### Landscapes of post-transcriptional regulation from the integrative analysis of large-scale gene expression datasets

With the aim of determining the extent of post-transcriptional regulation in human we analyzed large-scale^35^ RNA-seq data from 620 publicly available samples. Metadata of samples, which were generated with the removal of ribosomal RNA (rather than selection of polyA + RNAs), were retrieved through GEOmetadb queries. The actual expression data were obtained from the recount2 project^36^, following their homogeneous re-analysis in order to minimize the possibility of batch effects. Metadata were annotated with Onassis based on the CL and DOID ontologies, to determine cell line/tissue(s) and disease(s) associated with each dataset. The annotation procedure resulted in 26 tissue and 24 disease semantic sets. RNA-seq samples within each semantic set were then used to identify genes that were post-transcriptionally regulated for a given tissue and disease as described in^35^. Without the use of Onassis we could only have independently identified genes within each of the 620 samples. Rather, having assigned each sample to a semantic state, and having grouped samples associated to very similar semantic states, our analysis benefited by having multiple samples per condition (statistical power), and by having associated those samples to biologically meaningful, discrete, and related conditions (biological interpretability).

### A collection of regulatory elements from the integrative analysis of large-scale DNA methylation datasets

Low methylated regions (LMRs), with an average methylation of 30%, usually occur at distal regulatory elements^37^ and are associated with enhancers and regulatory factors driving the expression of cell identity genes^38,39^. Altered DNA methylation in these regions has been related to various diseases and contributes to cancer development^40–42^. We used Onassis functionalities to semantically annotate and organize a large DNA methylation dataset with the aim of identifying tissue or cell line specific LMRs, thus pointing to altered methylation patterns in specific disease conditions. We focused on the Marmal-aid repository^43^, which provides base-resolution information on the methylation of cytosines for 14584 samples from the human Infinium Illumina platform, profiling 450 K individual cytosines. As for the recount2 project, also Marmal-aid data were subjected to a re-analysis with homogenous conditions. We discarded the LMRs that could be found within hypomethylated promoters of expressed genes, and focused on 21209 LMRs that were distal from genes, since these represent potential regulatory elements. A manually curated table of samples’ metadata was included in Marmal-aid. We used Onassis to annotate those metadata with concepts belonging to the CL extended with concepts from Uber Anatomy ontology (UBERON)^44^ for anatomical parts. Eventually Onassis allowed to associate 13071 samples to 141 different cell type, which were eventually collapsed to a simplified set of 78 semantic states, each including one or more samples, i.e. sets of LMRs. We then proceeded to characterize tissue-specific LMRs. Finally, samples associated with each tissue, could also be associated to one or more disease conditions through the DOID Disease ontology. For each cell line/tissue, and for each disease condition, we focused on the LMRs that were present or absent compared to matched healthy samples. This analysis returned tissue specific catalogues of LMRs representing candidate regulatory elements that are epigenetically active in specific disease conditions. The ability of Onassis to assign samples to semantic conditions, and to determine their relatedness, was critical to identify the samples to be compared in their DNA methylation patterns (Supplementary File 1).

## Discussion

Onassis allows users to build large-scale structured datasets enabling the exploration of multiple conditions simultaneously. On one hand, this can allow to test hypothesis on previously published omics data. For example, searching for diseases that differ from the normal counterpart in terms of binding of a transcription factor. On the other hand, this can be instrumental for complementing and expanding in-house generated datasets. For example, a scientist working on a given regulatory factor, could study how its binding relate to the binding of other factors profiled by ChIP-seq in the same or very similar conditions^12^.

Onassis wraps different NLP tools and makes their functionalities conveniently available within the R/Bioconductor environment, where a number of packages could be used to perform additional analyses downstream the output of Onassis. The package focuses on and is particularly advantageous to the integrative analysis of omics data, but it can accommodate any corpus of text and ontology. Nowadays different projects such as Cistrome^32^ (for ChIP-seq data), recount2^36^ (for gene expression data), and Marmal-aid^43^ (for DNA methylation data), or public consortia such as ENCODE^45^ provide large datasets that have been processed with homogeneous pipelines and settings. These data are particularly suitable to the integrative analysis approaches implemented in Onassis, since batch effects due to alternative data analysis and normalization strategies are reduced.

In conclusion, Onassis greatly facilitates the identification and exploitation of publicly available datasets, thus promoting their re-use and providing biologists with the ability to analyze their own data within the context of the enormous wealth of knowledge nowadays available in public repositories.

## Supplementary information

Supplementary File1.

## Data Availability

Onassis is available as an R/Bioconductor package: https://bioconductor.org/packages/release/bioc/html/Onassis.html A step-by-step guide on the package functionalities - including the analysis of H3K27ac epigenomics data, the evaluation of Onassis performance, and the source code to reproduce the reported analyses - can be found in the package vignette: https://bioconductor.org/packages/release/bioc/vignettes/Onassis/inst/doc/Onassis.html Supplementary File 1 illustrates the application of Onassis to a large compendium of DNA methylation datasets.
