# Supplementary material for: Ontology-driven integrative analysis of omics data through Onassis
Source: Sci Rep. 2020 Jan 20;10:703. doi: 10.1038/s41598-020-57716-1 (PMC6971239; doi:10.1038/s41598-020-57716-1)
Supplement: Supplementary file 1 — Supplementary File1. [file 41598_2020_57716_MOESM1_ESM.zip › SupplementaryFile1.html]

Ontology driven integrative analysis of omics data through Onassis


# Ontology driven integrative analysis of omics data through Onassis

#### *Eugenia Galeota, Kamal Kishore, Mattia Pelizzola*

#### *12 december 2019*

### Introduction

Methylation of cytosines is an epigenetic modification of DNA (Pelizzola and Ecker 2011). Tissue specific regions are known to be hypo-methylated (Low Methylated Regions, LMRs). These regions show average methylation of 30% and occur at distal regulatory elements (usually enhancers). Altered DNA hypo-methylation patterns can be associated with various diseases and contribute to the development of cancer. We decided to focus on the LMRs present in various tissues and disease conditions within the Marmal-aid repository (Lowe and Rakyan 2013), consisting of 14584 human samples analyzed with the Infinium Methylation 450K array platform. The following document reports the code used to carry out the annotation and analysis of LMRs metadata and data. To run the code below the following libraries need to be installed and loaded in R:

- Onassis (Bioconductor)
- data.table (CRAN)
- DT (CRAN)
- TxDb.Hsapiens.UCSC.hg19.knownGene (Bioconductor)
- org.Hs.eg.db (Bioconductor)
- pheatmap (CRAN)
- parallel (CRAN)
- rGREAT (Bioconductor)

### Low methylated regions retrieval and definition

Base-resolution DNA methylation data represents the highest quality source of data in the field of DNA methylation. Currently, the most popular technology allowing to profile this mark at base-resolution is the Infinium Human Methylation 450K array.  
We reasoned that a low methylated cytosine from a 450K experiment could indicate the presence of an LMR. To minimize the likelihood of false positives, regions surrounding a low methylated cytosine were considered as LMRs if these overlapped with LMRS identified by whole genome bisulfite sequencing experiments (WGBS LMRs).

To this purpose, we collected 45 WGBS datasets from http://www.genboree.org/EdaccData/Current-Release/experiment-sample/Bisulfite-Seq/ to be used as a reference set of trustable LMRs. We used the methylSeekR Bioconductor package (Burger et al., 2013) to mask partially methylated domains, then the methylation levels of the remaining regions were smoothed over 3 consecutive CpGs in order to reduce the sampling noise. WGBS LMRs were defined as consecutive regions with at least 4 CpGs with smoothed methylation levels below 0.4. Simultaneously, array data were retrieved from Marmal-aid (Lowe and Rakyan 2013). Genomic regions surrounding a cytosine with methylation level < 0.3 and that overlapped at least one WGBS LMR were considered as potential LMRs These regions were further filtered according to their distance from the TSS of known genes as described in the code below.

```
load("./data/Infi_LMRs.Rdata")
```

```
# Finding distal regions in the potential LMRs list 

txdb <- TxDb.Hsapiens.UCSC.hg19.knownGene
EG2GS <- org.Hs.eg.db

downstream = 1000
upstream = 2000
gb <- transcripts(txdb, columns=c('tx_id','tx_name','gene_id')) #gene bodies annotation

suppressWarnings(prom <- promoters(txdb, upstream=upstream, downstream=downstream, 
                                   columns=c('tx_id','tx_name','gene_id')))        #promoters annotation

names(prom) <- prom$gene_id
names(gb) <- gb$gene_id

inds <- which(start(prom)<0)
if(length(inds)>0) start(prom)[inds]=1 

ind <- which(duplicated(gb$tx_name)==TRUE) #filtering out duplicated transcripts
if(length(ind) > 0) {
  gb <- gb[-c(ind)]
  prom <- prom[-c(ind)]
}

# cutting promoter regions downstream the TSS from genebody regions
shortinds <- which(width(gb) <= downstream)
gbNotProm <- gb[-shortinds]
plusStr <- which(as.logical(strand(gbNotProm) == '+'))
minusStr <- which(as.logical(strand(gbNotProm) == '-'))
start(gbNotProm[plusStr]) <- start(gbNotProm[plusStr]) + downstream
end(gbNotProm[minusStr]) <- end(gbNotProm[minusStr]) - downstream

# Filtering gene bodies and promoters from Marmal-aid samples
system.time(Infi_LMRs_intergenic <- mclapply(Infi_LMRs, function(sample_granges){
  gbInGR <- findOverlaps(query=gbNotProm, subject=GRmidpoint(sample_granges),
                         type='any', select='all')
  promInGR <- findOverlaps(query=prom, subject=GRmidpoint(sample_granges),
                           type='any', select='all')
  if(length(subjectHits(gbInGR))>0)
    sample_granges <- sample_granges[-subjectHits(gbInGR)]
  if(length(subjectHits(promInGR))>0)
    sample_granges <- sample_granges[-subjectHits(promInGR)]
  sample_granges
}, mc.cores=12))

names(Infi_LMRs_intergenic) <- gsub("_", "-", names(Infi_LMRs_intergenic))

# Removing if there are, the samples with 0 intergenic LMRs
lengths <- lapply(Infi_LMRs_intergenic, length)
Infi_LMRs_intergenic <- Infi_LMRs_intergenic[!names(Infi_LMRs_intergenic) %in% names(which(lengths==0))]
```

In the following plot we show the distribution of the number of LMRs int the 14584 samples available in Marmal-aid.

```
##    Min. 1st Qu.  Median    Mean 3rd Qu.    Max. 
##    1413    3877    4304    4733    5207   20816
```

The number of LMRs ranges from a few thousands to many thousands for a given sample, with some samples including almost all the identified LMRs. These were mostly associated to colorectal cancer. We also computed the number of samples in which a LMR can be found.

Supporting the hypothesis of tissue/cell type specificity, we noticed that only around 10% of the LMRs are shared across ~10000 samples, while most of them appear in a lower number of samples. A comparative analysis of these data cannot prescind from the correct stratification of samples into cell/tissue and disease sets. Furthermore, aggregating data by similar data descriptions is a fundamental step of the analyses to increase the reliability of the comparisons, where multiple samples could be considered as replicates.

### Semantic annotation of Marmal-aid samples’ metadata with concepts from biomedical ontologies using Onassis

From Marmal-aid we obtained the metadata table with sample descriptions, which was saved in a data table:

```
marmalaid_annotations <- readRDS("./data/marmalaid_annotations.rds")
```

Marmal-aid metadata were annotated with concepts belonging to the Cell ontology (CL) describing cell types, but also integrating anatomical parts from the UBERON ontology. Human Disease ontology (DOID) was subsequently used to match metadata with diseases.

CL releases/2015-06-17 in OBO format was retrieved from the NCBO bioportal and saved as cl.obo in the local file system.

Generic or frequent uninformative concepts were excluded after the annotation. Onassis method `annotate` was used to create the dictionary and annotate the metadata, while `filterconcepts` was used to exclude unwanted concepts.

```
#Mapping Marmalaid annotations to CL terms
marmalaid_annotations$Id <- gsub("-", "_", marmalaid_annotations$Id) 

cell_obo <- './cl.obo' #path of the CL obo file downloaded in the current working directory 

# Annotation of the metadata with CL terms using the best known parameter configuration
marmalaid_cells <- annotate(marmalaid_annotations[, c('Id', 'SAMPLE_NAME', 'LINEAGE', 'TISSUE', 'TISSUE_SUBTYPE' )], 'OBO', dictionary=cell_obo, d_synonymtype='ALL', SearchStrategy='CONTIGUOUS_MATCH', CaseMatch='CASE_IGNORE', Stemmer='PORTER', StopWords='NONE', OrderIndependentLookup='OFF', FindAllMatches='NO',  e_synonymtype='EXACT_ONLY')

#Definition of terms to exclude
terms_to_exclude <- c('tissue' , 'mesoderm', 'ectoderm', 'endoderm', 'cell','female organism', 'male organism', 'group', 'Homo sapiens' )


marmalaid_cells <- filterconcepts(marmalaid_cells, terms_to_exclude)
```

Onassis objects are returned by the method `annotate` which summarizes the semantic annotations of a given Marmal-aid sample by concatenating the unique concept identifiers in the ‘term\_id’ field and the unique concept names in the ‘term\_name’ field of the resulting table. In the following sections we will refer to these list of concepts as “semantic set” or “semantic class”.

Semantic sets obtained from the annotation process can be retireved calling the method `entities` on Onassis instances.

```
##                          sample_id                        term_id
## 2933                    GSM1079483                 UBERON_0000178
## 2185                    GSM1051527                 UBERON_0000178
## 11698 TCGA_44_6144_01A_11D_1756_05                 UBERON_0002048
## 2651                    GSM1051993                 UBERON_0000178
## 8227                     GSM795371 UBERON_0002107, UBERON_0002394
## 7545                     GSM886095                 UBERON_0000955
##              term_name
## 2933             blood
## 2185             blood
## 11698             lung
## 2651             blood
## 8227  liver, bile duct
## 7545             brain
##                                                                                           term_url
## 2933                                                 http://purl.obolibrary.org/obo/UBERON_0000178
## 2185                                                 http://purl.obolibrary.org/obo/UBERON_0000178
## 11698                                                http://purl.obolibrary.org/obo/UBERON_0002048
## 2651                                                 http://purl.obolibrary.org/obo/UBERON_0000178
## 8227  http://purl.obolibrary.org/obo/UBERON_0002107, http://purl.obolibrary.org/obo/UBERON_0002394
## 7545                                                 http://purl.obolibrary.org/obo/UBERON_0000955
##                 matched_sentence
## 2933             Blood, Mesoderm
## 2185             Blood, Mesoderm
## 11698                       Lung
## 2651             Blood, Mesoderm
## 8227  Endoderm, Liver, Bile duct
## 7545             Ectoderm, Brain
```

We obtained 141 unique semantics sets for 13071 samples.

The following plot reports the distribution of the number of samples in each semantic set.

### Semantic similarity of cell line/tissue semantic sets

Semantic similarity between semantic sets was calculated with the similarity functions provided by Onassis. The semantic similarity between couples of cell line/tissue semantic sets is the groupwise semantic similarity between their respective CL concepts. The “Best Match Average strategy” was used to group LIN pairwise semantic similarities between single concepts (Galeota and Pelizzola 2016). These are default options of the method sim applied to the entities of an Onassis object.

```
marmalaid_cells <- sim(marmalaid_cells) # Obtaining the semantic similarity matrix of CL semantic sets
```

CL semantic sets were clustered based on a distance calculated as “1 - groupwise semantic similarities” of couples of semantic sets.

In the following chart we show the distribution of semantic similarities between couples of semantic sets.

### Unification of similar semantic sets

Similar semantic set can be collapsed by cutting the hierarchical clustering tree at a given height. In our case, couples of semantic sets with similarity higher than 0.75 were merged into a single one, represented by the union of concepts in each one. Onassis automatically recomputes the groupwise semantic similarities between the newly obtained semantic sets.

```
collapsed_cells <- Onassis::collapse(marmalaid_cells, 0.75)
```

We obtained 78 semantic sets. Their clustering is represented by the following dendrogram with label sizes are proportional to the total number of samples in each semantic set.

### Building the network of LMRs and semantic sets

Onassis entities were used to assign single LMRs belonging to Marmal-aid samples to cell/tissue semantic sets. For each association we also annotated the corresponding number of Marmal-aid samples. This procedure builds a bipartite network with two types of nodes, LMRs and CL semantic sets connected by edges that are weighted by the number of samples.

```
# This code returns a list with couples of LMRs and semantic sets
# Each couple is weighted by the number of samples annotated with that semantic set and 
# containing that LMR

#Considering only the samples for which we have annotations
Infi_LMRs_intergenic <- Infi_LMRs_intergenic[which(names(Infi_LMRs_intergenic) %in% entities(collapsed_cells)$sample_id)]
Infi_LMRs_string  <- Infi_LMRs_string[which(names(Infi_LMRs_string) %in% entities(collapsed_cells)$sample_id)]
rownames(entities(collapsed_cells)) <- entities(collapsed_cells)$sample_id

#Creating a list of annotations for all the lmrs in all the samples
system.time(edges_lists <- sapply(names(Infi_LMRs_string), function(sample){
  lmrs_in_sample <- Infi_LMRs_string[[which(names(Infi_LMRs_string)== sample)]]
  cbind(lmrs_in_sample, rep(as.character(entities(collapsed_cells)[which(rownames(entities(collapsed_cells))==sample), c('short_label')]), length(lmrs_in_sample)) )
}))


#Creating a list of edges for the bipartite network weighted by sample numbers
edge_list <- do.call("rbind", edges_lists)
edge_list <- setDT(as.data.frame(edge_list))
colnames(edge_list)[2] <- 'annotation'
system.time(lmrs_2_cells <- edge_list[, .N, by = .(lmrs_in_sample, annotation)])
```

The list of unique LMRs - semantic set annotations has a total of 755959 associations for 21209 LMRs and 78 CL semantic sets.

The previously created list was used to fill the elements of a bipartite matrix (having as rows the semantic sets and as columns the LMRs) with the number of samples in which the LMR appears divided by the total number of samples in the corresponding semantic set. We also stored the same matrix, but with raw edge weights.

```
#Here we created a matrix where each row contains an annotation set and each column a LMR
unique_edge_list <- lmrs_2_cells
ptm <- proc.time()
bipartite_matrix <-  matrix(0, length(unique(unique_edge_list$annotation)), length(unique(unique_edge_list$lmrs_in_sample)))
bipartite_matrix_row <-  matrix(0, length(unique(unique_edge_list$annotation)), length(unique(unique_edge_list$lmrs_in_sample)))

rownames(bipartite_matrix) <- rownames(bipartite_matrix_row) <- unique(as.character(as.vector(unique_edge_list$annotation)))
colnames(bipartite_matrix) <- colnames(bipartite_matrix_row) <- unique(as.character(as.vector(unique_edge_list$lmrs_in_sample)))


system.time(apply(unique_edge_list, 1, function(edge){
  annot <- as.character(as.vector(edge[2]))
  lmr <- as.character(as.vector(edge[1]))
  tot_samples <- length(unique(entities(collapsed_cells)$sample_id[which(entities(collapsed_cells)$short_label==annot)]))
  bipartite_matrix[which(rownames(bipartite_matrix)==annot), which(colnames(bipartite_matrix)==lmr)] <<- as.numeric(edge[3])/tot_samples
  bipartite_matrix_row[which(rownames(bipartite_matrix)==annot), which(colnames(bipartite_matrix)==lmr)] <<- as.numeric(edge[3])
}))

print(proc.time() - ptm)
```

The introduction of semantic annotations and semantic similarities within the dataset of LMRs allowed us to test the hypothesis of tissue/cell line specificity of the LMRs. Based on this hypothesis in fact, rates of overlap between semantic sets should increase according to the semantic similarity.  
The overlap rate of two semantic sets is the percentage of LMRs of the first semantic set that can also be found in the second semantic set.

```
lmrs <- unique(as.character(as.vector(lmrs_2_cells$lmrs_in_sample)))
annotations <- unique(lmrs_2_cells$annotation)
number_of_annotations <- length(annotations) - 1
overlaps_m <- matrix(NA, nrow=length(annotations), ncol=length(annotations))
rownames(overlaps_m) <- colnames(overlaps_m) <- annotations

for(i in 1:number_of_annotations) {
  sclass1 <- as.character(as.vector(annotations[i]))
  lmrs1 <- unique(lmrs_2_cells$lmrs_in_sample[which(lmrs_2_cells$annotation==sclass1)])
  j = i + 1
  for(k in j:length(annotations)){
    sclass2 <- annotations[k]   
    lmrs2 <- unique(lmrs_2_cells$lmrs_in_sample[which(lmrs_2_cells$annotation==sclass2)])
    overlaps_m[i, k] <- length(which(lmrs1 %in% lmrs2)) / length(lmrs1)
    overlaps_m[k, i] <- length(which(lmrs2 %in% lmrs1)) / length(lmrs2)
  }
}
diag(overlaps_m) <- NA
rownames(overlaps_m) <- colnames(overlaps_m) <- annotations
```

The distribution of the overlap ratios within increasing intervals of semantic similarity between semantic sets is reported here.

We confirmed the general increase in the overlap of semantics sets for similarity values becoming higher.

#### Identification of Semantic set specific LMRs

We decided to score LMRs based on their cell line or tissue specificity. Following the approach often used to asses the specialization levels of species and plants in pollination networks (Dormann, n.d.), we computed the Shannon partner diversity, quantifying the diversity of LMRs with respect to semantic annotation sets. Lower values of the measure indicate higher tissue/cell line specificity.

```
shannon <- function(x, base=exp(1)) {# shannon's diversity index
    prob <- x/sum(x)
    -sum(prob*log(prob, base=base), na.rm=TRUE)
 }

logbase <- exp(1)
 
col_sums <- colSums(bipartite_matrix_cells)
bipartite_matrix_cells <- bipartite_matrix_cells / col_sums
specificities <- apply(bipartite_matrix_cells, 2, shannon, base=logbase)
```

To compute a threshold for the specificity we generated 10000 null models using the function `rd2table`. Briefly, each null model assigns to each semantic set the same number of LMRs (column sums) and for each LMR the same number of samples (row sums), but scatters the interactions across all semantic annotation sets (Patefield algorithm). This procedure tends to create LMRs that are very general. To create the 10000 null models we ran the following source code in parallel on a computer cluster.

```
null_matrices_list <- r2dtable(1000, rowSums(bipartite_matrix_cells), colSums(bipartite_matrix_cells))
```

We computed the specificity indexes for the null models LMRs.

```
null_scores <- lapply(null_matrices_list, function(null_matrix){
  col_sums <- matrix(rep(colSums(null_matrix), nrow(null_matrix)), nrow(null_matrix), byrow=T)
  null_matrix <- null_matrix / col_sums
  specificities <- apply(null_matrix, 2, shannon, base=logbase)
})
```

p values were obtained from the following code:

```
specificity_matrix <- do.call('rbind', null_scores)
p.values <- sapply(seq(1, length(specificities), 1), function(lmr_index){
  true_specificity <- specificities[lmr_index]
  null_column <- specificity_matrix[, lmr_index]
  length(which(null_column<true_specificity)) /length(specificities)
})
names(p.values) <- names(specificities)
```

### Annotating diseases

Having scored LMRs in terms of tissue/cell line specificity, we decided to consider the disease context, to search for differences among different disease conditions or healthy. Human Disease Ontology version doid/releases/2019-08-22/doid-non-classified.obo was obtained from https://raw.githubusercontent.com/DiseaseOntology/HumanDiseaseOntology/master/src/ontology/doid-non-classified.obo. The same configuration of parameters used for tissues and cell lines was used also for diseases. disease parameter of the method `annotate` was set to true to find also ‘Healthy’ occurrences. As for tissues, we decided to exclude general concepts.

```
disease_obo <- './data/doid-non-classified.obo'
marmalaid_annotations <- readRDS("./data/marmalaid_annotations.rds")

#Mapping Marmalaid annotations to DO diseases
marmalaid_annotations$Id <- gsub("-", "_", marmalaid_annotations$Id)
marmalaid_annotations$DISEASE_SUBTYPE <- gsub("\x92\xe4ξ", " ", marmalaid_annotations$DISEASE_SUBTYPE)

#Definition of terms to exclude
terms_to_exclude <- c('disease')

#Annotation of the field DISEASE with DOID
marmalaid_disease <- annotate(marmalaid_annotations[, c('Id', 'DISEASE', 'DISEASE_SUBTYPE' )], 'OBO', dictionary=disease_obo, d_synonymtype='ALL', SearchStrategy='CONTIGUOUS_MATCH', CaseMatch='CASE_IGNORE', Stemmer='PORTER', StopWords='NONE', OrderIndependentLookup='OFF', FindAllMatches='NO',  e_synonymtype='EXACT_ONLY', disease=TRUE)


marmalaid_disease <- filterconcepts(marmalaid_disease, terms_to_exclude)
```

Onassis annotation resulted in 84 for 10648 samples.

### Merging tissue and disease annotations

With the method `mergeonassis` diseases were associated to Marmal-aid samples and their corresponding the cell/tissue semantic sets.

```
onassis_tissue_disease <- mergeonassis(collapsed_cells, marmalaid_disease)
```

### Creating the score matrix

A binary matrix indicating the presence of LMRs in Marmal-aid samples was obtained from the initial list of LMRs. Rows of the binary matrix are the distal LMRs, while columns are Marmal-aid samples. Entries of the matrix are set to 1 if the LMR on the row is present in the sample on the column.

```
Infi_LMRs_string <- Infi_LMRs_string[which(names(Infi_LMRs_string) %in% entities(onassis_tissue_disease)$sample_id)]
unique_lmrs <- unique(unlist(Infi_LMRs_string))
samples <- names(Infi_LMRs_string)
score_matrix <- matrix(0, nrow=length(unique_lmrs), ncol=length(samples))
rownames(score_matrix) <- unique_lmrs
colnames(score_matrix) <- samples

for(i in 1:length(samples)){
  print(i)
  score_matrix[Infi_LMRs_string[[i]], i] <- 1 
}
```

To compare specific LMRs in tissue/cell semantic sets we decided to use Onassis `compare` function. The set of LMRs was restricted to those with p-value obtained from specificities <= 1e-5. Semantic sets were compared according to their overlap. The overlap rate of a semantic disease set d1 with a semantic disease set d2 is given by the number of the common LMRs divided by the number of LMRs in d1. Multiple columns (samples) of the binary LMRs score matrix, which are assigned to the same disease semantic set were summarized for each LMR using the majority rule. The function that we define executes the following operations:

- for each disease semantic set retrieves the columns of the score matrix associated to the samples annotated with that disease
- creates a binary vector of the same lenght of specific LMRs and for each LMR sets the corresponing value to 1 if at least half of the samples contain that LMR
- computes the overlap of couples of disease semantic sets within each cell line/tissue semantic set

The `compare` method automatically retrieves the tissue and disease information from the entities of the Onassis object and for each tissue/cell semantic set where at least two different diseases or healthy can be identified, applies the overlap function. A list with overlap rates of couples of diseases within each semantic set is returned.

```
#Filtering LMRs by p.values 
filtered_lmrs <- specificities[which(p.values<=1e-5)] 

# Associating the list of GSM ids to each couple of tissue/cell line and disease semantic sets 
gsms <- aggregate(entities(onassis_tissue_disease)$sample_id ~ entities(onassis_tissue_disease)$short_label_1 + entities(onassis_tissue_disease)$term_name_2, data=entities(onassis_tissue_disease), c)
specific_score_matrix <- score_matrix[names(filtered_lmrs),]

# Personalized overlap function to use in the compare method. The function takes two parameters
# x and y can be either a vector indicating presence or absence of specific LMRs or a subset of the score matrix where each column corresponds to a sample
overlap_function <- function(x, y){
  print('computing overlap')
  pval <- NA
  # for each LMR we set value to 1 if the LMR is present in at least half of the samples in the first semantic set
  if(is.matrix(x)){
    x <- apply(x, 1, function(row){
       as.numeric(names(which.max(table(row))))
    })
  }
  # for each LMR we set value to 1 if the LMR is present in at least half of the samples in the second semantic set
  if(is.matrix(y)){
    y <- apply(y, 1, function(row){
     as.numeric(names(which.max(table(row)))) 
    })
  }
  #overlap of two semantic sets
  overlap_x_y <- length(which(x==1 & y==1)) / length(which(x==1))
  return(list(statistic=overlap_x_y, p.value=NA)) 
}

#Applying the compare method to obtain the overlap of 
diseases_overlap <- compare(onassis_tissue_disease, score_matrix=as.matrix(specific_score_matrix), by='col', fun_name='overlap_function')
```

Heatmaps of the overlaps follow

Heatmaps of overlap

### GREAT annotation of tissue/disease semantic sets

Within each tissue/cell line semantic set, for each disease or healthy condition, enrichments of genomic regions corresponding to the top 500 specific LMRs were obtained using the package rGREAT.

```
#This file allows to carry out enrichment of the regions 
# in the GRanges previoyusly filtered and annotated with GRannotate of compEpiTools
# and 

specific_score_matrix <- score_matrix[names(filtered_lmrs),]
entities(onassis_tissue_disease) <- entities(onassis_tissue_disease)[which(! entities(onassis_tissue_disease)$sample_id =='GSM1051878'),]
gsms <- aggregate(entities(onassis_tissue_disease)$sample_id ~ entities(onassis_tissue_disease)$short_label_1 + entities(onassis_tissue_disease)$term_name_2, data=entities(onassis_tissue_disease), c)

gsms <- gsms[which(gsms[,1] %in% names(diseases_overlap)),]
occurrence <- 1
great_enrichments <- apply(gsms, 1, function(tissue_disease){
       list_of_gsms <- as.character(unlist(tissue_disease[3]), recursive=TRUE)
       score_matrix_subset <- as.matrix(specific_score_matrix[, list_of_gsms], ncol=length(list_of_gsms))
        list_of_lmrs <- rownames(score_matrix_subset)[rowSums(score_matrix_subset)>0]
        list_of_lmrs <- sort(filtered_lmrs[list_of_lmrs], decreasing=FALSE)
        list_of_lmrs <- names(list_of_lmrs[1:500])
        lmrs_ranges <- GRanges(list_of_lmrs)
        #The submitGreatJob function carries out the enrichment of the regions considering basalPlusExt: mode 'Basal plus extension'.
        # Gene regulatory domain definition: Each gene is assigned a basal regulatory domain of a minimum distance upstream and downstream of the TSS #(regardless of other nearby genes). The gene regulatory domain is extended in both directions to the nearest gene's basal domain but no more than #the maximum extension in one direction.
        #adv_upstream: proximal extension to upstream (unit: kb)  <--- default is 5
        #adv_downstream: proximal extension to downstream (unit: kb) <-- default is 1
        #adv_span: maximum extension (unit: kb) <- defult is 1000 = 1mb for distal elements
        job <- submitGreatJob(lmrs_ranges, species = "hg19")
        table_of_annotations <- getEnrichmentTables(job, ontology = availableOntologies(job) )
        occurrence <<- occurrence + 1
        table_of_annotations
})
```

With the following code we created hatmaps of the enrichments for each disease state and tissue/cell line semantic set. Enriched terms with Raw p-value <= 1e-3 were considered for each disease.

```
#List to contain enrichments
enrichment_list <- list()
k <- 1
# For each tissue
for(tissue_name in names(diseases_overlap)){
  #Retrieval of the diseases associated to the tissue
  diseases <- rownames(diseases_overlap[[tissue_name]])
  great_indexes <- c()
  # For each disease findd the enrichments from GREAT
  for(disease in diseases){
    great_index <- which(gsms[,1]==tissue_name & gsms[,2]==disease)
    great_indexes <- c(great_indexes, great_index)
  }
  enrichments_subset <- great_enrichments[great_indexes]
  names(enrichments_subset) <- diseases
  list_of_matrices <- list()
  #Separating the annotations by 7 different annotation databases
  for(i in 1:7){
    db_matrices <- lapply(enrichments_subset, '[[', i)
    db_matrices <- lapply(db_matrices, function(single_matrix){
      single_matrix <- single_matrix[, c(2, 8)]
      single_matrix <- single_matrix[which(single_matrix[,2]<=1e-3),]
    })
    unique_values <- unique(unlist(lapply(db_matrices, '[[', 1)))
    heatmapDataMatrix <- matrix(0, nrow=length(unique_values), ncol=length(enrichments_subset))
    rownames(heatmapDataMatrix) <- unique_values
    colnames(heatmapDataMatrix) <- diseases
    for(j in 1:length(db_matrices)){
      disease <- db_matrices[[j]]
      row_names <- disease[,1]
      column <- diseases[j]
      heatmapDataMatrix[match(row_names, rownames(heatmapDataMatrix)), column] <- -log10(as.numeric(as.vector(disease[,2])))
    }
    list_of_matrices[[i]] <- heatmapDataMatrix
  }
  names(list_of_matrices) <- names(enrichments_subset[[1]])
  enrichment_list[[k]] <- list_of_matrices
  k <- k + 1
}
names(enrichment_list) <- names(diseases_overlap)
```

Heatmaps

# References

Dormann, Carsten F. n.d. “How to Be a Specialist? Quantifying Specialisation in Pollination Networks.”

Galeota, Eugenia, and Mattia Pelizzola. 2016. “Ontology-Based Annotations and Semantic Relations in Large-Scale (Epi)genomics Data.” *Briefings in Bioinformatics*. https://doi.org/10.1093/bib/bbw036.

Lowe, Robert, and Vardhman K. Rakyan. 2013. “Marmal-Aid - a Database for Infinium Humanmethylation450.” *BMC Bioinformatics* 14 (1): 359. https://doi.org/10.1186/1471-2105-14-359.

Pelizzola, Mattia, and Joseph R. Ecker. 2011. “The Dna Methylome.” *FEBS Letters* 585 (13): 1994–2000. https://doi.org/10.1016/j.febslet.2010.10.061.
